# Considerations on Milk, and Particularly That of Nurses, More Especially in Regard to Its Good or Bad Nutritive Qualities, and the Changes to Which It Is Subject

**Published:** 1838-07

**Authors:** 


### ART. XIV.

*Du Lait, et en particulier de celui des Nourrices, considéré sous le rapport de ses bonnes et de ses mauvaises Qualités nutritives, et de ses Alterations.* Par le Dr. AL. DONNÉ, &c.—*Paris,* 1837. 8vo. pp. 66.

*Considerations on Milk, and particularly that of Nurses, more especially in regard to its good or bad Nutritive Qualities, and the Changes to which it is subject.* By Dr. AL. DONNÉ.

IT is much to be desired that, when all the solids and fluids of the body, healthy and diseased, have been determined to consist of certain globules of various size, shape, and consistence, some useful information may be associated with such discovery. Hitherto we can say little more than that observers have been contented with stating what they have seen; some of them having acquired such amount of credit as belongs to their possessing a good microscope and an acute sense of sight: others, less fortunate, having either described forms for which they have been indebted to fertile imaginations, or such as were the distorted images of bad glasses or equally bad eyes. M. Donné has been some years known to the profession, for his minute enquiries on different physiological subjects, and has published essays on Saliva, Mucus, and the Spermatic Animalculæ. We notice preferably the present paper, because it is not only the latest, but that which bears most directly on practical medicine. There are certainly both remarks and facts in it which appear to be worthy of our attention. The condition of human milk in relation to the health of children has not been sufficiently attended to. When the apparently hopeless states of disease from which children are sometimes recovered by a change of milk are remembered, as well as the uncertainty as to the nature of these morbid conditions, it becomes a question, well worthy of an attempt at solution, whether there is any perceptible quality of the milk by which these may be explained. It cannot be said that the essay of Dr. Donné is very satisfactory on this point, but it is a beginning of the enquiry, and contains hints which may be easily adopted by others who are willing to examine the subject for themselves. We have satisfied ourselves, by examination, of the correctness of his description of milk in a mature state; and, taking this as a standard, the detection of deviations cannot be difficult, and may be in some instances very useful and important. Dr. Donné remarks, that inferences as to the suitableness of milk from the healthy or unhealthy appearance of the female are very fallacious; and that the external appearance of the fluid is often very good, when the microscope detects a morbid condition; so that, without this mode of examination, our knowledge of milk must necessarily be very imperfect. In addition to the microscope, Dr. Donné employs a few very simple reagents: these we shall mention in the course of our analysis. The essay chiefly treats of the milk of women in reference to the nourishment of children, and to this part we shall limit our notice.

The first chapter is devoted to a *Microscopical Examination of Milk.* It has been long known that milk contained globules. In their normal condition, these globules are of various sizes, perfectly spherical, with black and regular borders; and they swim freely in a fluid in which no other particles are suspended. The composition of milk may be thus stated:—A liquid, holding in solution sugar of milk, salts, a small quantity of fatty matter and caseum, and suspending globules of different sizes, consisting of butter, and soluble in ether. If some cow's milk or ass's milk be filtered, and the liquid which passes the filter be examined, scarcely any globules will be found, and such as do exist are extremely small. This fluid, however, contains a large quantity of caseum. The globules remain in the filter with the cream, which appears to consist almost exclusively of them. If this cream is agitated in a tube with ether, the globules are completely dissolved. Hence it follows that the globules belong to the fatty constituent of the milk. Concentrated ammonia has no effect on these globules, and caustic solutions of potass and soda act upon them with great difficulty, and only after a considerable time. At the end of twenty-four hours, the majority of the globules still exist in a solution of potass or of ammonia, and the solution requires to be heated to act upon some of them.

The second chapter notices the first kind of alteration in milk; *its Mucous State, and the Persistence of this Fluid in the Condition of Colostrum.* The author has examined human milk previous to delivery, at the time of delivery, and during the changes which occur in it, until it has arrived at its perfect condition. It is known as a general fact that the early milk differs from that which is subsequently formed, and that its qualities are adapted to the necessities of the infant. The more evident qualities of the colostrum are as follows: it is a yellowish fluid, consisting of two parts, one serous, the other viscid; the consistence of the latter is that of a syrup. If allowed to stand in a vessel, a layer of considerable thickness is formed on its surface, of a yellowish colour more or less deep, which is the cream containing a large quantity of butter; if this is taken off, and the fluid is heated, a second, and even a third, is formed. The same phenomena occur during the three days which succeed delivery; with this difference, that the layer becomes less and less thick the nearer to the fourth day after delivery (p. 20;) but, if the colostrum is examined by the microscope, it is found to possess other characteristics, much more marked, and more worthy of consideration.

We have already mentioned the appearance of perfect milk. The colostrum is very different. It contains some real milk globules, but they are irregular and disproportioned; some of them appear like large oleaginous drops, and cannot be termed globules: this is evidently the imperfectly elaborated butter; that which rises to the surface of the colostrum and forms the yellow layer. The majority of the other globules in the colostrum are very small, and look like dust in the midst of the fluid: these globules, instead of swimming separately, are mostly connected together by a viscid matter; so that, when they are moved about over a glass plate, they separate in small agglomerated masses, instead of rolling one over the other, as is the case in perfect milk. In addition to this, the colostrum contains particles which have no relation with the common globules of milk: some of these are very small, their diameter being about one hundredth of a millimêtre; others are many times larger. These are but slightly transparent, of a yellowish colour and granular appearance; they seem to be composed of a multitude of small grains, connected together or enclosed in a transparent envelope; and frequently there is found within one of these little masses a true globule of milk. Dr. Donné imagines that these *granular bodies* (as he terms them) may consist of a fatty matter and peculiar mucus. They are not soluble in alkalies, but, as is the case with the true lactic globules, they disappear in ether; and after the evaporation of this solvent, small acicular crystals remain upon the glass.

Of the above description, the author has given, in a plate, representations with which it very accurately accords.

This condition of the milk continues, almost unchanged, to the end of the milk fever: then the liquid gradually changes; the number of granular bodies diminishes day by day; the lactic globules acquire a more regular and definite form, and, without being all of the same size, they differ less in this respect than was previously the case. At the same time these globules, previously united "*en masse*," and associated in a confused manner by means of a viscid substance, separate themselves, become isolated, and move in the fluid, quite independent of each other. These modifications do not always take place in the same period of time; but some traces of the primitive condition of the milk are appreciable twenty-four days after delivery, in very healthy women. This is the rule; to which there are exceptions, to be presently noticed.

It has been shown that pure milk does not become viscid when treated with concentrated ammonia: but ammonia renders the colostrum glairy, fibrous, and tenacious. This is a character common to the colostrum and to purulent matter. Both the colostrum and milk are alkaline: Dr. Donné has never found them otherwise. This is contrary to the observation of others; but, after repeated examinations, made expressly to determine the fact, and being aware of the contrary opinion expressed by others, the author can but state that, in his examinations, the red turnsol paper has uniformly become of a blue colour. The human milk, that of cows, asses, and goats, are all alkaline; and this is the case at all times of the year, and under all circumstances of food, season, &c. The opinion which has been formed of the acidity of milk, Dr. Donné considers as arising from its not having been tested immediately after having been drawn from the animal.

Sometimes milk retains the characteristics of colostrum beyond the ordinary period; occasionally for months, or during the whole time of suckling: and this milk, to common observation, appears as white, as consistent, as when healthy. The condition of the fluid can, consequently, be only ascertained by microscopic examination. Some milk remains in the state of colostrum a long while; and, in comparing this condition with that of the female by whom it was formed, she has been found very frequently lean and ill-nourished. The following is a case in illustration:—A young woman was confined with her second child, July 23, 1836; she was apparently very healthy. On the 1st of August, the milk was abundant and its aspect healthy, except that it was somewhat viscid. The child was quite healthy and well formed, but it frequently refused the breast without any appreciable cause. For twenty days after delivery, the milk remained in the condition of colostrum, as above described, but its colour was normal, its consistence as in the healthy state, and externally this milk appeared as healthy as that of the best nurses. Eighteen days after delivery, the child had diarrhœa; the milk did not change its character; and, twelve days subsequently, the child died, having gradually become emaciated. The former child by the same female died at the age of five months. Dr. Donné merely mentions this fact without wishing to infer any necessary connexion between the deaths of the children and the condition of the mother's milk: he regards it, however, as a fact which well deserves attention. The microscopic examinations which the author has made of the milk of asses and goats, during the state of pregnancy, after delivery, and until the milk has arrived at its mature condition, have shown a great analogy between these and that of women; with one exception, that the granular bodies belonging to the colostrum of woman are much less abundant in that of animals. These experiments also further confirmed the fact that the external appearance of milk is not a fair test of its actual qualities.

The third chapter is devoted to *Pathological Changes in Milk.* The mammæ, it is well known, become occasionally engorged during the period of suckling. Such a case is related where the engorgement occurred the eighth day after delivery. The milk presented the appearance of a number of globules connected together by a mucous substance; became viscid on the addition of ammonia, and contained a certain number of granular bodies. Similar changes were observed in another case, in which the breast became tumid and painful after delivery. From what has preceded, it is considered as established that the agglomeration of globules of milk and the presence of granular bodies, are the signs of milk which is either imperfectly formed or not of good quality. This modification takes place either in consequence of a lesion of the gland, or of a change of the lacteal secretion depending on a general disturbance of the health, as in the following case:—A young woman, eighteen years of age, eight days after delivery, was seized with colic and fever, which created the suspicion of metro-peritonitis. As long as this condition continued, the milk was full of granular bodies, and became viscid when ammonia was added to it. Two applications of leeches to the abdomen calmed the pain, and convalescence was established. From this moment the milk acquired its normal character. There was still some agglomeration among the globules, but no foreign bodies could any longer be seen.

But in addition to these changes in milk, it may be mixed with actual pus, and the most scrupulous attention might not discover the mixture: Dr. Donné has seen several instances of this. The globules of milk are perfectly spherical, transparent, and with black borders; those of pus are spotted, jagged, and opaque. Alkaline solutions completely dissolve the purulent globules, and leave the milky globules untouched. Ether dissolves the latter, but has no action on the former. From an abscess in the mammary gland, the pus may of course escape; and, in doing so, mix with the milk: but Dr. Donné has also found that the pus may become mixed with milk in the lactiferous vessels, and escape with it from the nipple; a fact of considerable importance where it is the common practice to encourage the infant in sucking a breast in which pus has been formed. In one case, where the fluid escaping from the nipple presented globules both of milk and pus, Dr. Donné correctly diagnosticated an abscess which was not previously detected. That the author has looked in vain for venereal matter in milk will not require the long explanation which he has given of the fact. In animals, Dr. Donné has found blood in milk, but he has never met with a similar case in women.

The last chapter considers the *proportion of the nutritive Elements of Milk;* and it is stated that the quantity of fatty matter in the same species of milk is generally in proportion to the quantity of the other solid elements of this fluid; so that the richness of milk may be inferred with tolerable accuracy from the number of globules which it contains. The milk of nurses may be in fault, as well from superabundance as from a deficiency of nutritious parts. The diameter of the globules appears to increase the longer the time since the delivery; but this sign is of no use to ascertain the age of milk.

